# Onshore Wind Energy Development Causes Localized but Lasting Shifts in Plant Community Composition and Function

**DOI:** 10.1002/ece3.73916

**Published:** 2026-06-29

**Authors:** Lukas Seifert, Katrine Sivertsen, Audun Rugstad, Bente J. Graae, Roel May, Dagmar Hagen

**Affiliations:** ^1^ Norwegian University of Science and Technology Trondheim Norway; ^2^ Norwegian Institute for Nature Research Trondheim Norway

**Keywords:** disturbance, ecosystem restoration, mitigation, plant community composition, road construction, ruderal species, vegetation impacts, wind energy

## Abstract

Wind power plants are frequently placed in natural ecosystems, but their impacts on plant communities are rarely considered. Therefore, it is unknown how far potential impacts extend into adjacent vegetation and how long they persist. To address this, we surveyed vegetation at different distances to roads at three wind power plants in Norway that were commissioned 4, 12, and 19 years ago. We then used Grime's CSR strategies to document functional shifts in plant community composition and Ellenberg Indicator Values (EIVs) to identify the abiotic gradients driving these shifts. We found that shifts in plant community composition were related to road distance and time since disturbance. At the youngest site, the proportion of plants with ruderal strategies was significantly increased within 10.4 m of roads, effectively expanding the footprint of roads by more than two‐fold. At the oldest site, this impact was reduced to 2.8 m, suggesting that the original stress‐tolerant communities recovered at a rate of 0.5 m per year. Increased ruderality near roads was associated with plant communities indicating higher nutrient availability and more reactive soils. This study provides novel knowledge regarding the spatial and temporal impact of wind energy development on plant communities. As road construction appears to shift community composition toward ruderal dominance by increasing nutrient availability, we recommend keeping road‐ and construction areas to a minimum. Overall, this can reduce the footprint of wind power plants and ensure that the transition to renewable energy does not come at the expense of ecosystems.

## Graphical abstract

The effects of wind power plants on surrounding vegetation remain poorly understood. Vegetation surveys at three Norwegian wind power plants commissioned 4, 12, and 19 years ago showed that roads caused shifts in plant functional strategies toward species with ruderal strategies. Although stress‐tolerant communities recovered over time, these shifts effectively increased the footprint of roads by at least two‐fold. Overall, this highlights the importance of careful road planning and post‐construction management.

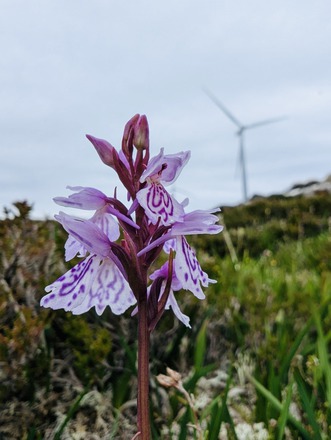

**Abbreviations**
CLV: concurrent latent variable
CSR: competitor, stress‐tolerator, ruderal
CWM: community‐weighted mean
EIV: ellenberg indicator value
LA: leaf Area
LDMC: leaf dry matter content
NMDS: non‐metric multidimensional scaling
SD: standard deviation
SLA: specific leaf area
WPP: wind power plant

## 1 Introduction

The transition from fossil fuels to renewable energy sources is crucial for meeting increasing energy demands while reducing greenhouse gas emissions. More than half of the global energy supply is expected to come from renewables by 2040, with wind energy contributing up to 25% (International Energy Agency 2023). In Norway, the development of onshore wind energy has expanded rapidly over the past 25 years. According to the Norwegian Water Resources and Energy Directorate (NVE), 65 wind power plants (WPPs) operate across the country. Over two‐thirds of them were built within the past decade (NVE 2023). Despite this rapid expansion, proposals for new WPPs are often met with concerns regarding their environmental consequences (Kraft and Kraft 2025). While wildlife conflicts are relatively well studied, impacts on vegetation remain poorly understood, particularly regarding how far they extend into adjacent areas and how long they persist. However, since vegetation underpins key ecosystem functions such as soil stability (Dahanayake et al. 2024), carbon storage (Lange et al. 2015), and habitat provision (Rutten et al. 2015), resolving vegetation impacts is a crucial step toward reducing the broader ecological footprint of wind energy.

Wind energy development affects vegetation through two main mechanisms. First, wind turbine operation can modify microclimatic conditions, including wind exposure, precipitation, and shading. This may alter vegetation growth and survival (Diffendorfer et al. 2022; Kaffine 2019). Second, the installation of wind turbines is dependent on the construction of access roads and turbine pads, which generate direct disturbances by clearing vegetation, compacting soils, and altering microtopography and hydrology (Gunn et al. 2002). Because roads constitute the largest fraction of land used by WPPs (Denholm et al. 2009), they may play a central role in shaping vegetation responses to wind energy development. Apart from immediate impacts during construction, roads can cause ongoing disturbances through nitrogen deposition from vehicles and mineral inputs from gravel particles (Bignal et al. 2007). They have also been shown to facilitate the spread of invasive plant species within WPPs (Keehn and Feldman 2018; Villarreal et al. 2019).

Secondary succession theory describes how plant communities change over time following disturbance events (Horn 1974). In early successional stages, communities are typically dominated by fast‐growing species adapted to high light levels, shifting nutrient availability, and physically unstable conditions (Douma et al. 2012). In later stages, community composition shifts depending on species interactions, changing resource availability, and environmental filters that constrain species dispersal and recruitment (Brown and Cahill JR. 2020; Meiners et al. 2015). Grime's CSR strategy theory classifies plant species based on how they respond to varying levels of disturbance and resource availability (Grime 1974, 1977, 2006). It distinguishes between three primary functional strategies: Competitors (C), which dominate in stable, resource‐rich environments; stress‐tolerators (S), which are adapted to harsh conditions and low resource availability; and ruderals (R), which thrive in disturbed habitats and usually dominate early successional stages. Here, we use the term *ruderality* to refer to the degree to which a plant community is dominated by species with ruderal strategies.

Another approach to understanding how disturbances shape plant communities is through Ellenberg Indicator Values (EIVs). EIVs provide species‐specific optima along key abiotic gradients such as light availability, soil moisture, and nutrient status (Ellenberg et al. 1992). Traditionally they are used to infer site conditions from vegetation composition, but they can also be applied in reverse to investigate how abiotic factors filter species during community assembly in succession (Di Biase et al. 2023; Mastrogianni et al. 2023). In contrast to CSR, EIVs offer a more direct lens on species' abiotic preferences, allowing for the detection of shifts in environmental optima. For example, vegetation clearing during road construction may increase light availability by creating canopy gaps, thereby favoring species with high light values. EIVs thus reveal how disturbances shape plant communities by promoting species whose abiotic preferences align with the altered conditions (Bartelheimer and Poschlod 2016).

Here, we use CSR strategies to characterize functional shifts in plant community composition following WPP development at three sites along the Norwegian coast, and EIVs to identify the abiotic gradients driving these shifts. Specifically, we ask: (1) To what extent does WPP development increase ruderality in adjacent vegetation? (2) Does ruderality in operational WPPs decline over time? (3) Which abiotic gradients are associated with shifts in ruderality and plant community composition in WPPs? and (4) Which species are most sensitive to disturbances from roads and wind turbines?

We expect that: (1) Ruderality increases near roads and turbine pads as a result of vegetation removal and soil disturbance, (2) Oder sites show signs of recovery, characterized by a decline in ruderality and a shift toward species with competitive or stress‐tolerant strategies, (3) Plant communities at WPPs are shaped by increased light availability resulting from vegetation removal and reduced soil moisture caused by drainage, and (4) Wetland species adapted to stressful conditions are displaced near roads and turbines.

By answering these questions, we respond to a pressing knowledge gap regarding the ecological consequences of wind energy development. First, by determining how far ruderal species extend into adjacent vegetation, we provide a functional measure of its footprint—that is, the spatial reach on plant community composition and the time needed for recovery. Second, by identifying the underlying abiotic drivers and plant species that are favored or displaced, we inform potential mitigation or restoration measures. Overall, this knowledge supports wind energy development that is not only renewable but also sustainable for the ecosystems it occupies.

## 2 Materials and Methods

### 2.1 Study Sites

**FIGURE 1:**
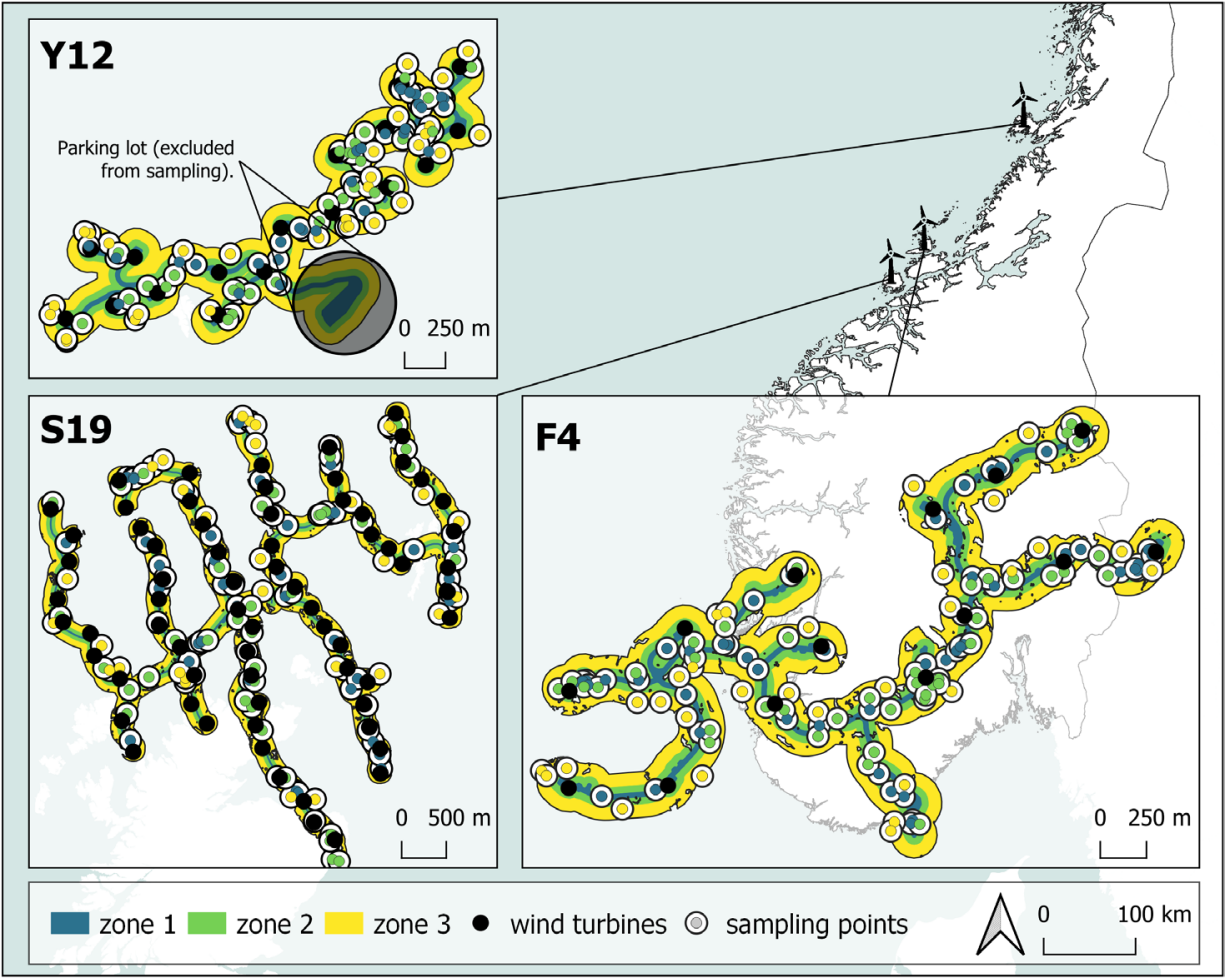
Locations of the Smøla (S19), Ytre Vikna (Y12) and Frøya (F4) wind power plants. The insets show the study design: At each site, we established three zones at different distances to roads and surveyed vegetation at 30 random points per zone: 0–10 m (zone 1), 10–50 m (zone 2), and 50–120 m (zone 3).

To compare plant community composition across WPPs, we selected three sites of similar vegetation type, topography, and landscape context (Figure 1). This selection was guided by Landsat 8 and 9 satellite images (EROS 2020) and CORINE land cover maps (EEA 2020). All sites are located on islands (Smøla, Ytre Vikna, and Frøya) along the west coast of central Norway (63.4°–64.9° N; 8.0°–10.9° E) and characterized by open, low‐growing vegetation with wetland elements, underlain by hard bedrock (Table 1). Climatic conditions are similarly cool and humid. However, we purposely chose WPPs that differ in age in order to use space‐for‐time substitution to assess recovery time. To reflect these differences, we labeled each site based on its location and the number of years elapsed since WPP commissioning at the time of data collection (2024): Smøla (19 years, S19), Ytre Vikna (12 years, Y12), and Frøya (4 years, F4). Note that S19 entered operation in 2002 but was expanded by 48 turbines in 2005 (Table 1).

**TABLE 1:** Characteristics of the three study sites.

Category	S19	Y12	F4
Location	Smøla	Ytre Vikna	Frøya
WPP commissioning	2002 (2005^a^)	2012	2020
Number of turbines	20 + 48^a^	17	14
Turbine height (m)	70	64	112
Turbine diameter (m)	76–82.4	71	136
Elevation (m asl)	10–40	60–150	20–80
Yearly precipitation (mm)	1586	2302	1178
Potential sampling area (km^2^)	6.35	1.69	2.37
Geology	Gabbro, migmatite, amphibole gneiss, granodiorite, diorite	Granitic gneiss, grandioritic gneiss	Granite
Vegetation type	Sparse vegetation and wetland	Sparse vegetation and wetland	Sparse vegetation and wetland
Dominant species	*Calluna vulgaris* , *Empetrum nigrum, Pleurozium schreberi*	*Calluna vulgaris, Empetrum nigrum, Racomitrium lanuginosum*	*Calluna vulgaris, Potentilla erecta*, *Racomitrium lanuginosum*

^a^ S19 was expanded in 2005 by an additional 48 wind turbines.

### 2.2 Sampling Design

In May 2024, we visited S19 and observed that the largest differences in plant community composition were concentrated within 10 m of roads. Simultaneously, remote sensing studies indicate that vegetation impacts can extend beyond 100 m from turbines (Liu et al. 2023). Based on these findings, we implemented a systematic study design in QGIS version 3.28 (QGIS Development Team 2025), following four steps. First, we defined a sampling area extending 120 m from roads and turbine pads to capture the full range of potential impacts (Figure 1). Second, we divided the sampling area into three zones relative to roads and turbine pads to ensure sufficient coverage of the highly disturbed zone we observed at S19: 0–10 m (zone 1), 10–50 m (zone 2), and 50–120 m (zone 3). Third, we excluded water bodies from the sampling area based on an area resource map of Norway (Heggem et al. 2019). Finally, we randomly allocated 30 points in each zone, spacing them at least 10 m apart to reduce spatial autocorrelation. Importantly, since the three zones represent initial estimations based on our site visit and literature values, the actual level of disturbance at each point might not accurately be reflected by the zone it is located in. Therefore, we also obtained the absolute distance between each point and the nearest road and turbine from QGIS. In total, we established 270 points (3 sites × 3 zones per site × 30 points per zone) for vegetation sampling (Figure 1).

### 2.3 Vegetation Sampling

**FIGURE 2:**
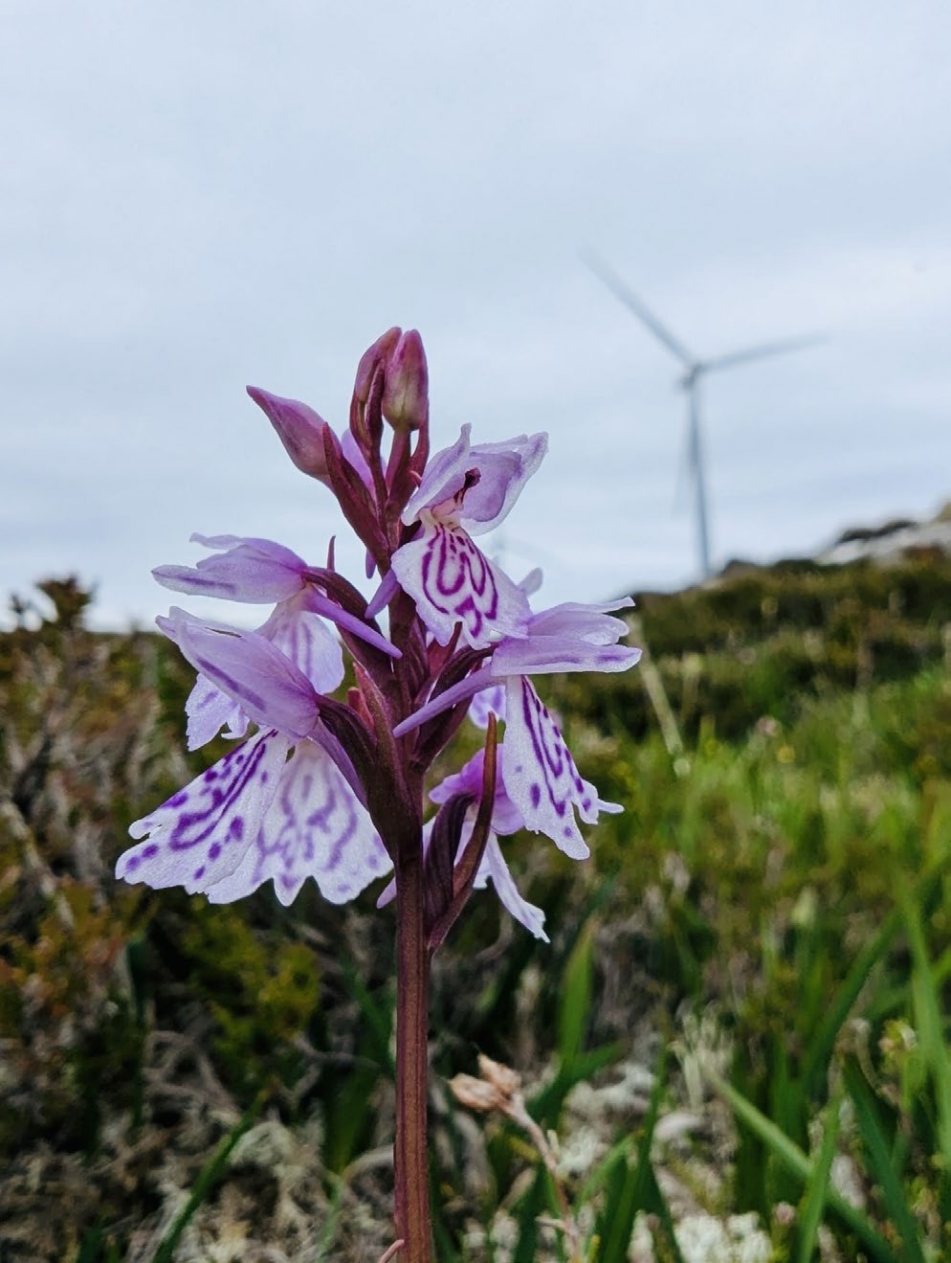
*Dactylorhiza maculata* (Heath spotted orchid) in the foreground of a wind turbine at Smøla wind power plant (S19). Photo: Matthieu Maugis.

We conducted vegetation surveys in June and July 2024. They were coordinated with the WPP operators although no permits were required. First, we located sampling points with a handheld Etrex 30 GPS unit (Garmin Ltd., Olathe, USA) and established sampling plots by placing vegetation frames directly over the points. Vegetation frames measured 1 × 0.5 m, divided into twenty equally sized subplots. Then we estimated the cover of bare rock, open water, bare soil, and loose gravel. Bare soil and loose gravel were recorded as a proxy for recent disturbance and vegetation gaps. Bare rock and open water were estimated to ensure representative vegetation coverage under undisturbed conditions. If their combined cover exceeded 20%, the plot was moved 1 m away from the road along a north–south axis and step three was repeated. All cover estimates (except open water) were later used to explain variations in plant community composition and species abundance (Section 2.5). Finally, we recorded all vascular plant species (Figure 2) and bryophyte species and quantified their abundance as frequency (i.e., the number of subplots in which they occurred), following species nomenclature provided by the Norwegian Biodiversity Information Centre (Artsdatabanken). Since we could not reliably identify them, we recorded nine bryophyte taxa and three vascular taxa (*Euphrasia, Hieracium, and Taraxacum*) at genus level.

### 2.4 Linking Functional Strategies and Abiotic Gradients

#### 2.4.1 CSR Scores

We used CSR scores to assess shifts in plant functional strategies in response to WPP development. CSR scores indicate how plant species adapt to different levels of disturbance and resource availability (Grime 1974). Based on them, each species can be positioned within a triangular space representing its adaptation to productive environments (competitive species), resource‐limited or stressful conditions (stress‐tolerant species), and disturbance (ruderal species). Consequently, each species is characterized by three proportional scores C, S, and R which sum to one and reflect its location within the CSR triangle (Ricotta et al. 2023).

For most species, we obtained CSR scores directly from the global dataset compiled by Pierce et al. (2017) and its extension for northern species by Novakovskiy et al. (2021). This provides a standardized way to estimate CSR scores without having to measure functional traits on site. For species without readily available CSR scores (*Huperzia selago, Sagina procumbens, Narthecium ossifragum, Cirsium heterophyllum, Carex binervis, and Salix aurita*), we calculated them using StrateFy (Pierce et al. 2017). StrateFy is a globally calibrated Excel‐tool that estimates CSR scores based on leaf area (LA), specific leaf area (SLA), and leaf dry matter content (LDMC) by using empirically established relationships of leaf trait variation and ecological strategies. To use StateFy, we obtained the relevant trait data (LA, SLA, and LDMC) from the TRY database (Kattge et al. 2020), which is a global repository of plant trait measurements. Although most of the species (e.g., *H. selago, N. ossifragum*, and *S. aurita* ) are associated with nutrient‐poor bogs where trait expression is expected to be comparable across sites, their CSR scores should be interpreted as approximations of general strategies rather than site‐specific trait expressions.

Finally, we aggregated species' CSR scores into a community‐level representation of functional strategies by calculating community‐weighted means (CWMs). CWMs represent the average score of a given strategy in a community (here plot), weighted by the relative abundance of species possessing that strategy (i.e., plots that are dominated by species with ruderal strategies have a high ruderality). CWMs can be useful for quantifying community structure and change (Garnier et al. 2004; Quétier et al. 2007), which we visualized using ternary diagrams provided from the R package *ggtern* (Hamilton and Ferry 2018).

#### 2.4.2 Ellenberg Indicator Values

We used Ellenberg indicator values (EIVs) to identify which abiotic gradients were driving the observed shifts in plant community composition. EIVs are semi‐quantitative ordinal scores that represent species' ecological optima along key abiotic gradients (Ellenberg et al. 1992). They are assigned based on observed species distribution and expert calibration (Tichý et al. 2023). For example, moisture values range from 1 for species associated with very dry conditions to 12 for submerged aquatic species. Here, we were interested in EIVs for light availability (L), moisture (F), reactivity (R), and nitrogen availability (N), as we expect them to play an important role in secondary succession. We obtained EIVs from the PLANTATT database for vascular plants (Hill et al. 2004) and from the BRYOATT database for bryophytes (Hill et al. 2007) and used them in three complementary ways. First, we fitted CWM EIVs to the NMDS ordination to visualize how abiotic gradients structured plant communities (Section 2.5). Second, we related CWM EIVs to ruderality scores to assess how ruderal strategies align with local environmental conditions (Figure A1). Third, we incorporated species‐specific EIVs into a fourth‐corner model to quantify how species' abiotic preferences vary with distance to roads and wind turbines and among study sites (Section 2.5).

### 2.5 Statistical Analyses

#### 2.5.1 Visualizing Plant Community Composition

All statistical analyses were conducted in R version 4.5.1 (R Core Team 2021). To visualize differences in plant community composition, we performed non‐metric multidimensional scaling (NMDS) using the R package *vegan* (Oksanen et al. 2017). The NMDS was based on species frequency data from plots containing at least four species. We calculated a dissimilarity matrix using the Bray‐Curtis distance, which is appropriate for community data and sensitive to species abundances (Bray and Curtis 1957). The final NMDS used three dimensions and multiple random starts (trymax = 250) and produced a stress value below 0.2, indicating an acceptable level of ordination fit (Dexter et al. 2018). To statistically test differences in plant community composition between zones, we used PERMANOVA with zone as a fixed factor. To identify the abiotic factors that play a role in community composition, we applied the *envfit* function, including substrate cover estimates (Section 2.3) and EIVs (Section 2.4) at the plot level.

#### 2.5.2 Determining the Spatial and Temporal Reach of Ruderal Species

To determine how far ruderal plant species extend into adjacent vegetation and how long they persist over time, we used segmented regression analysis. We did this because the decline in ruderality with distance from roads was abrupt, and segmented regression can detect abrupt changes by estimating breakpoints along the distance axis that mark shifts in community response (Tomal and Ciborowski 2020). We fitted a linear model with distance to roads or turbines as the predictor and CWM CSR scores as the response. Then, we applied the *segmented* function of the R package *segmented* (Muggeo and Muggeo 2017) to estimate one breakpoint per site. Consistent with our predefined zones (Section 2.2), we specified an initial breakpoint guess of 10 m. From the model output, we extracted the breakpoints and their 95% confidence intervals. To test whether breakpoints marked a significant shift in community composition, we conducted a *t*‐test comparing CWM CSR scores before and after the breakpoint.

#### 2.5.3 Understanding the Effect of Abiotic Factors

To understand which abiotic factors drive shifts in plant community composition in responses to WPP disturbance, we performed a fourth‐corner analysis on the association between EIVs and disturbance‐related variables, including the distance to the nearest wind turbine, the zone identity, and the study site as a proxy of time since disturbance. We fitted the fourth‐corner model using the R package *gllvm* (Niku et al. 2021). The model included community‐wide effects of the disturbance‐related variables with a random intercept for each species and a nested interaction effect with the species' EIVs. We chose the oldest site (S19) and least disturbed zone (zone 3) as reference categories as we expect them to host the least disturbed plant communities. Because many species had a high proportion of zero‐observations and full presence, the model was fitted using the ordered beta distribution as response distribution (Korhonen et al. 2024) and five starting iterations to ensure convergence. We confirmed model validity by visually inspecting randomized quantile Dunn‐Smyth residuals (Dunn and Smyth 1996).

#### 2.5.4 Species' Sensitivity to Disturbances From Roads and Turbines

To test how individual plant species respond to proximity to roads and wind turbines, we used a concurrent latent variable (CLV) model (Van Der Veen et al. 2023). CLVs offer three main advantages: (1) The degree to which the environmental variables explain differences in species composition between sites can be explored, for example by variance partitioning, (2) Individual species responses can be estimated as a function of the environmental and species coefficients of the latent variables, and (3) As CLVs can be fitted within the generalized linear mixed model‐based framework, standard errors for species and latent variable coefficients can be used to assess the significance of each predictor in explaining changes in species abundance. We fitted our CLV model to all species occurring in more than six plots (*n* = 59) using the R package *gllvm* (Niku et al. 2021). To account for the stratified sampling design, we included zone, distance to road, and their interaction effect as predictors for the latent variables. We also included an unstructured, sample‐specific random effect to account for differences in total abundances among plots. Again, the model was fitted using an ordered beta distribution and validated using randomized quantile Dunn‐Smyth residuals.

## 3 Results

### 3.1 Recently Disturbed Sites Hosted More Species

The disturbance levels at each site generally mirrored recovery times, particularly close to roads. In zone 1, the coverage of bare soil was highest at F4 (32%, SD = 36%), followed by Y12 (8%, SD = 15%), and S19 (0%). All differences between sites were statistically significant (pairwise Wilcoxon rank‐sum tests, BH‐adjusted *p* < 0.01). Across all sites, we recorded a total of 111 vascular plant species and 35 bryophytes, but total species richness was higher at F4 (118 species) compared to Y12 (107) and S19 (89). Average species richness, however, was higher at Y12 (14.8 species per plot) than at F4 (12.9, *p* < 0.01) and S19 (12.5, *p* < 0.01). Finally, species richness was similar across zones at S19 and Y12 but increased from 10.8 in zone 1 to 13.8 in zone 2 and 14.1 in zone 3 at F4 (*p* = 0.01).

### 3.2 Ruderality Declined With Distance From Roads

**FIGURE 3:**
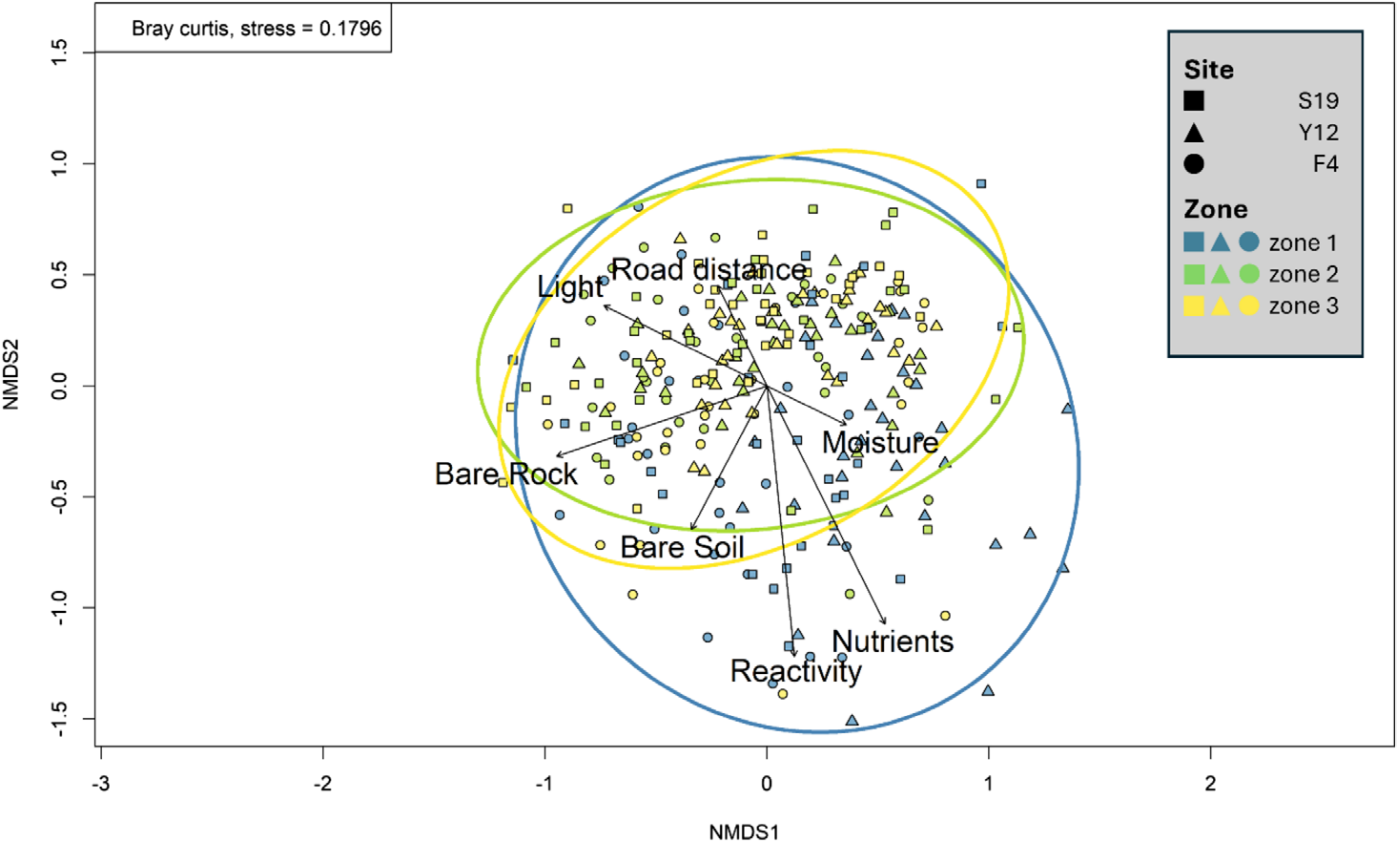
Non‐metric dimensional scaling (NMDS) of sampling plots based on species abundance across the Smøla (S19), Ytre Vikna (Y12), and Frøya (F4) wind power plant (*n* = 270). Colored ellipses represent 95% confidence intervals of plots grouped by zones. Arrows represent abiotic drivers that are significantly correlated with plant community composition. Arrow length indicates the strength of correlation, and direction shows the gradient along which the community changes.

Plant communities were similar between sites but differed between zones (Figure 3). While plant communities in zones 2 and 3 had a substantial overlap in species composition, plant communities in zone 1 only partially resembled those from zones 2 and 3. Overall, zone identity explained 5.4% of the variation across plots, indicating modest but significant differences in plant community composition depending on the zone (PERMANOVA, F = 7.54, R^2^ = 0.054, *p* < 0.01).

**FIGURE 4:**
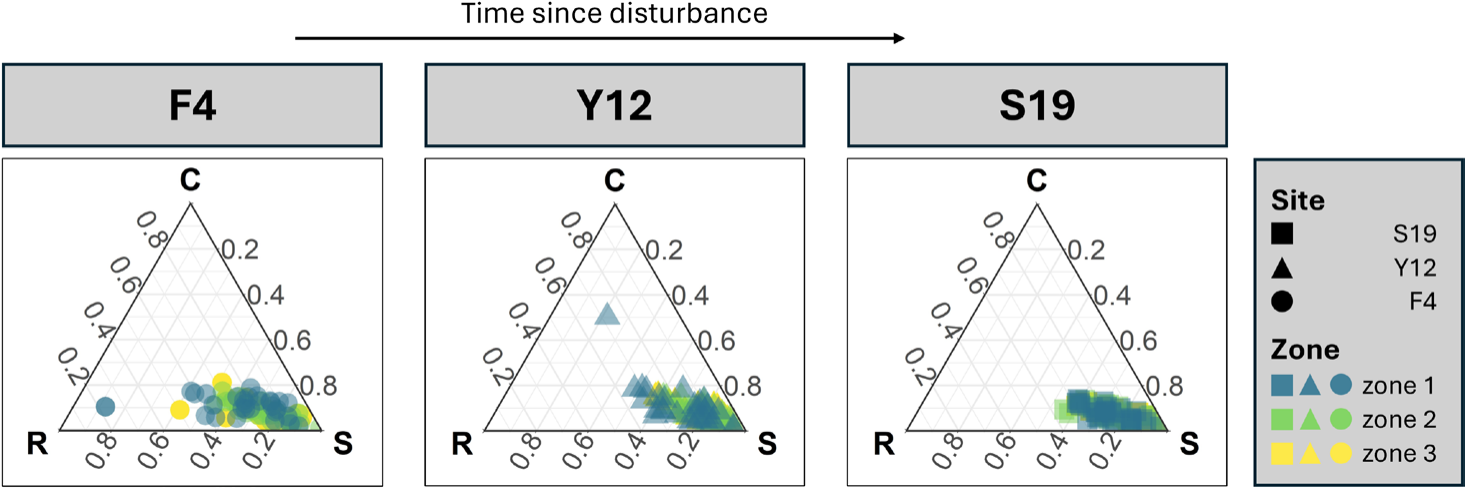
CSR scores for each plot in the Smøla (S19), Ytre Vikna (Y12) and Frøya (F4) wind power plants, shown separately for each site and grouped by distance to roads based on zones: 0–10 m (zone 1), 10–50 m (zone 2), and 50–120 m (zone 3). Each point represents the community‐weighted mean (CWM) of competitive (C), stress‐tolerant (S), and ruderal (R) strategies.

**FIGURE 5:**
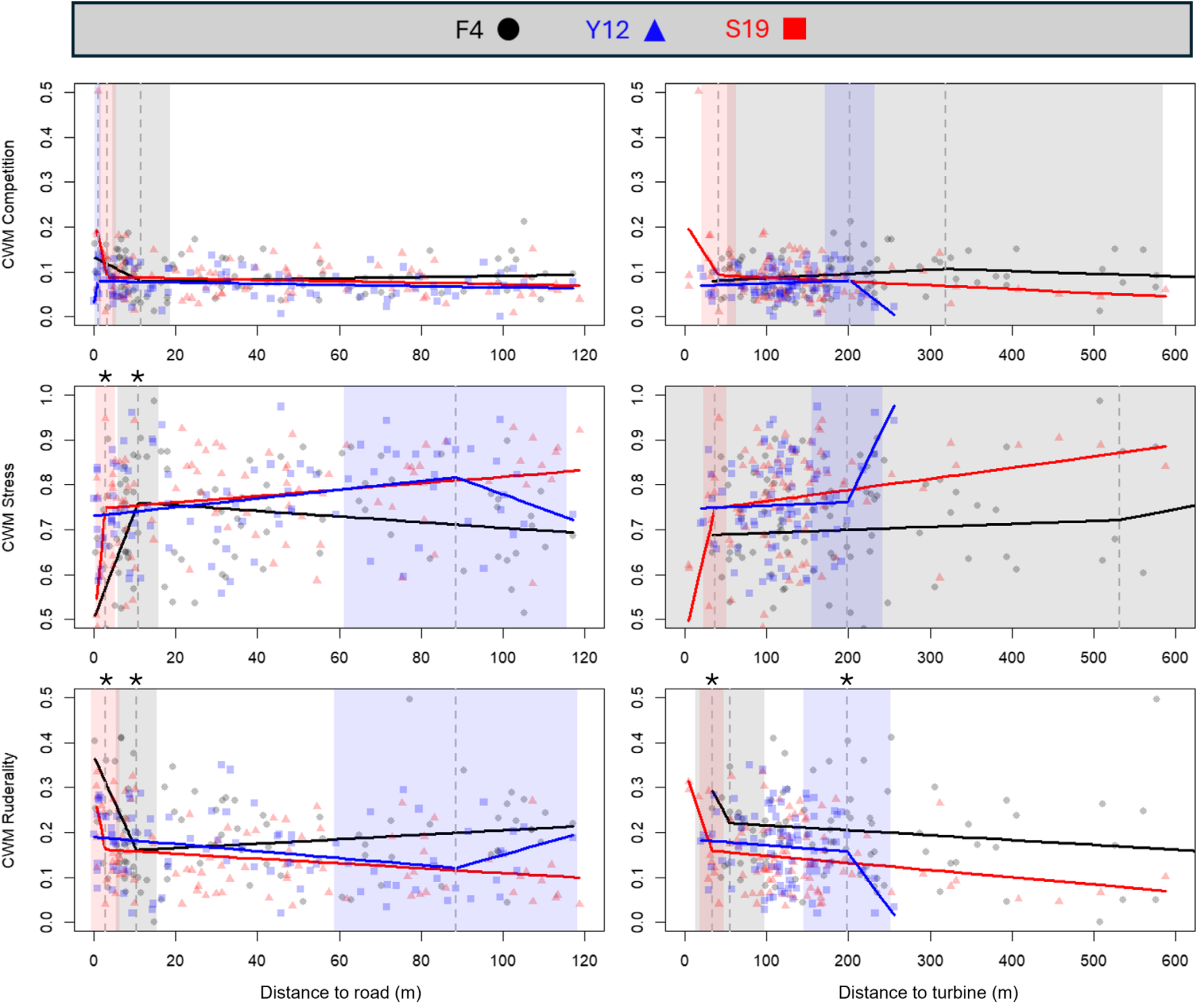
Segmented regression of community‐weighted mean (CWM) CSR scores and distance to the nearest road (left) and wind turbine (right) at the Frøya (F4), Ytre Vikna (Y12), and Smøla (S19) wind power plants. Data points represent individual plots (*n* = 270). Solid lines show site‐specific regression fits, with the dashed vertical lines marking estimated breakpoints in meters. Shaded areas denote 95% confidence intervals around the breakpoint estimates. Breakpoints where CWM values differ significantly from plots on either side (*t*‐test, *p* < 0.05) are marked with a star.

At all three sites, plant communities were dominated by species with stress‐tolerant strategies, with an average stress‐tolerance between 0.76 (S19, SD = 0.10) and 0.70 (F4, SD = 0.15) (Figure 4). Y12 was dominated by stress‐tolerant strategies across all zones, but F4 and S19 exhibited a shift toward ruderal strategies along the S‐R axis, particularly in zone 1 (Figure 4). Segmented regression identified the distances at which these shifts occurred by estimating breakpoints along the road distance axis (Figure 5). Ruderality was significantly higher near roads, with breakpoints at 10.4 m at F4 (*p* = 0.05) and 2.8 m at S19 (*p* = 0.01). Conversely, CWM stress‐tolerance was significantly lower near roads, with breakpoints at 10.8 m at F4 (*p* = 0.02) and 2.8 m at S19 (*p* = 0.03). At Y12, a similar shift from ruderality toward stress‐tolerance was suggested beyond 80 m from roads, but it was not statistically significant (*p* = 0.50).

### 3.3 Plant Communities Near Roads Indicated High Nutrient Availability

**FIGURE 6:**
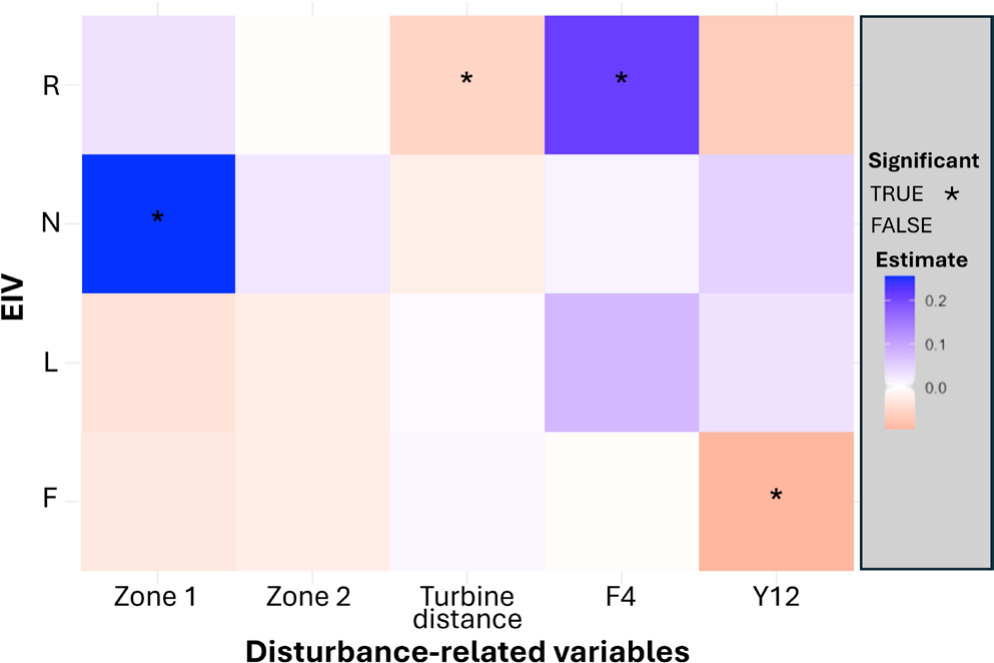
Association terms between EIVs and disturbance‐related variables from the fourth corner model. Variables include distance to road based on zone (1 and 2) compared to zone 3; distance to nearest wind turbine; and site F4 (Frøya) and Y12 (Ytre Vikna) compared to S19 (Smøla). Stars mark association terms which were significant at the 95% confidence level.

Plant communities in zone 1 were dominated by species associated with nutrient‐rich and more reactive soils, as well as slightly drier conditions (Figure 3, Figure 6). Communities in zones 2 and 3 were dominated by species adapted to higher light availability. Whereas all EIVs were associated with community composition (*p* < 0.01), the effects of nutrient availability, reactivity, and light were stronger than those of moisture (Figure 3). Ruderality was positively correlated with nutrient availability and reactivity but negatively correlated with moisture and light (Figure A1). Nutrient levels as indicated by EIVs were significantly higher in zone 1 compared to zones 2 and 3 (Figure 6). Additionally, reactivity values were negatively associated with the distance to wind turbines but were higher for communities at F4 compared to S19.

### 3.4 One in Six Plant Species Occurred Exclusively Near Roads

**FIGURE 7:**
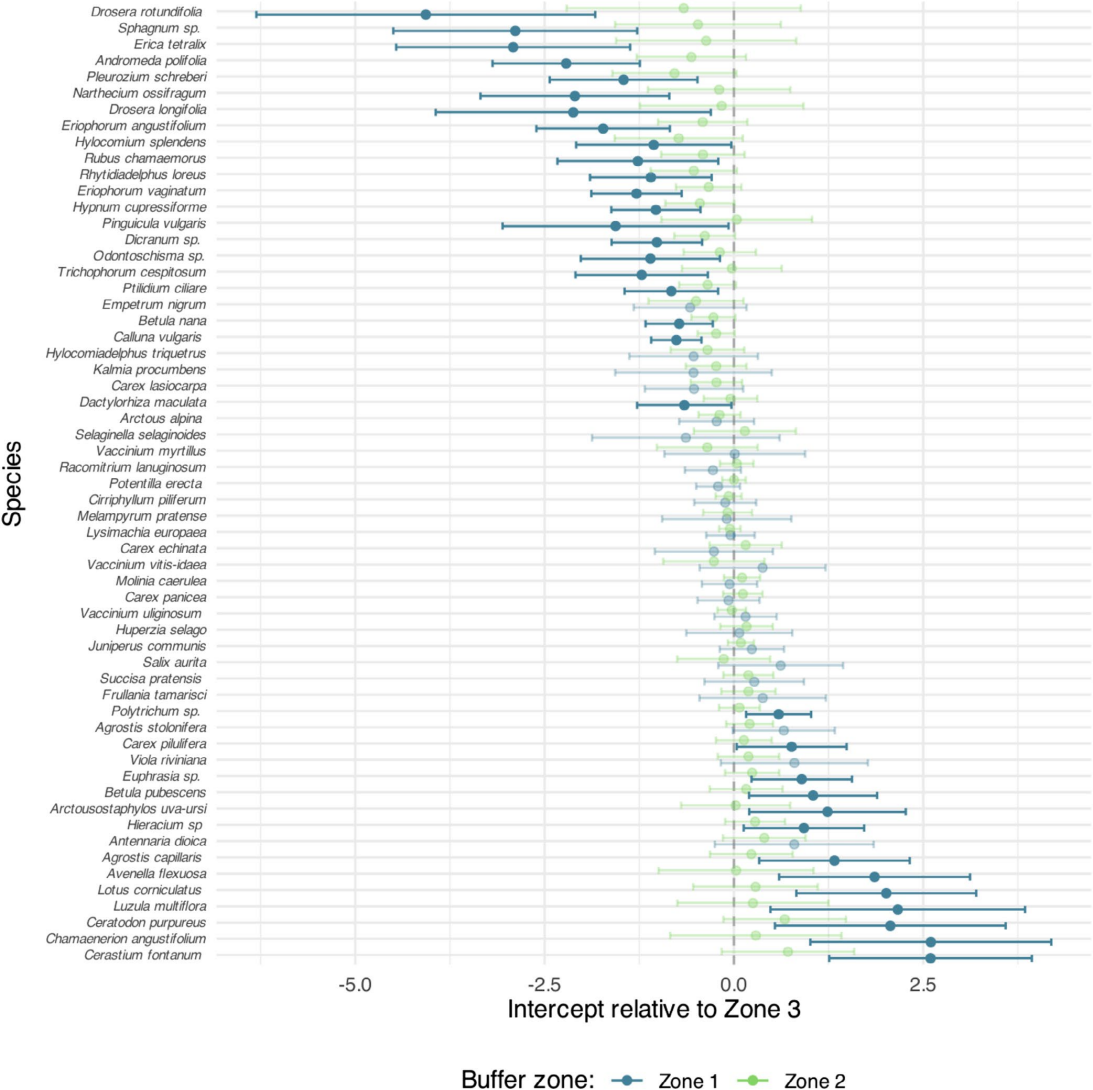
Species‐specific coefficients for the effect of distance to road by intercept in zone 1 and 2 compared to zone 3.

Of the 146 species surveyed, 23 occurred exclusively in zone 1 (Table A1). Species abundance varied with road distance, substrate cover, and site identity. Species that were more abundant near roads were, for example, *Chamaenerion angustifolium* , *Avenella flexuosa* L., and *Cerastium fontanum* Baumg. (Figure 7, Figure A2). Species that were less abundant near roads included *Erica tetralix* (L.), *Drosera rotundifolia* (L.), and *Sphagnum* mosses. Species that increased in abundance near roads were generally associated with higher percentages of bare soil and rock and vice versa (Figure A3). For example, *Cerastium fontanum* was less abundant in plots that were far from roads, but more abundant in plots with a high cover of bare soil or rock. Finally, *Empetrum nigrum* (L.), *Vaccinium myrtillus* (L.), and *V. vitis‐idaea* (L.) were significantly less abundant at F4 compared to S19 (Figure A4).

## 4 Discussion

### 4.1 Highlights

In this study we showed that roads associated with WPPs caused localized changes in plant community composition and function. Ruderality was significantly increased near roads, while the proportion of species with stress‐tolerant strategies declined. Although the spatial extent of ruderal takeover decreased over 15 years, ruderality remained elevated near roads even two decades after construction. Ruderality was positively associated with plant communities indicating higher nutrient availability and more reactive soils and negatively associated with moisture and light conditions. Potential differences in abiotic conditions were also reflected in species' responses to road proximity, together with substrate cover and site identity.

### 4.2 Ruderal Takeover and Successional Recovery

Ruderality was significantly higher within 10.4 m of roads at F4, but only within 2.8 m of roads at S19. Interpreting the two sites as points along a temporal gradient, and assuming similar initial disturbance, this suggests that the distance over which roads promote species with ruderal strategies decreased by 7.6 m in 15 years (or approximately 0.5 m per year). Beyond the disturbed roadsides, stress‐tolerance was the dominant strategy, aligning with the notion that vegetation recovery toward stable communities involves a functional shift to stress‐tolerant or competitive species (Ricotta et al. 2023; Caccianiga et al. 2006). Species with competitive strategies typically establish under conditions of low abiotic stress and high resource availability (Choler et al. 2001; Zanzottera et al. 2020). Our sites, however, were characterized by nutrient limitation, waterlogging, and short growing seasons, all of which would favor stress‐tolerators. Since the recovery of stress‐tolerant communities was still incomplete even nearly 20 years after commissioning, this provides evidence that wind energy development causes localized but lasting changes in plant community composition.

Interestingly, we did not detect a significant increase in ruderality along roads at Y12, despite its intermediate age. This is likely due to limited initial disturbance on rocky outcrops where machinery could not remove vegetation or compact soils. Since mortality is a key driver of successional change (Smith et al. 2022), low disturbance intensity would have restricted ruderal species establishment. The low ruderality at Y12 might hence reflect site‐specific constraints rather than effects of time since disturbance alone. To distinguish between them, we suggest long‐term monitoring of vegetation within the same WPPs, as it is challenging to find WPPs that are constructed either at the same time or in similar environments. Similarly, it remains difficult to disentangle the individual effects of roads and wind turbines, as they do not occur independently within WPPs. Although the study design was optimized for roads, the results should be interpreted as reflecting the combined influence of roads and turbines.

### 4.3 Potential Nutrient Deposition Along Roads

Road construction can disturb soils, expose mineral substrates, and increase nutrient availability along roads (Zhao et al. 2007). Our results support the importance of this mechanism, as EIVs for nutrients were the strongest predictors of plant community composition, especially in zone 1. After construction, nutrients from gravel roads and road dust can accumulate in adjacent soils and influence vegetation composition in otherwise nutrient‐poor environments (Müllerová et al. 2011; Gill et al. 2014). Nutrient‐ and dust depositions along roads has been shown to decline within a few meters of the disturbance (Jaźwa et al. 2016; Trombulak and Frissell 2000), also in peatlands (Li et al. 2023).

Vegetation may also be impacted by air pollution from vehicles. For example, oak and beech forests showed increased defoliation, insect damage, and poorer crown condition close to roads, extending up to 100 m and closely matching NO_2_ concentration profiles (Bignal et al. 2007). Although car access is permitted for visitors during weekends at Y12 and for locals at S19, traffic intensity at our study sites is generally low. The observed shifts in plant community composition are therefore likely driven by nutrient inputs associated with road construction and deposition rather than air pollution.

Despite not directly measuring nutrient loads, we showed that plant communities in disturbed areas had a high affinity for nutrients based on EIVs. In north‐west Europe, high levels of nitrogen deposition could increase the dominance of grass species on heathlands at the expense of dwarf‐shrubs (Britton et al. 2003). Our study reveals how this shift could manifest along roads within WPPs. *Chamaenerion angustifolium* and *Avenella flexuosa* were among the species most positively related to road proximity. *A. flexuosa* can outcompete *Calluna vulgaris* (L.) under conditions of increased nitrogen contents (Hofland‐Zijlstra and Berendse 2010). On sandy soils in oceanic climates, *C. vulgaris* can regain dominance, but it is usually suppressed by *A. flexuosa* on peaty soils (Britton et al. 2003). As a generalist with flexible strategies (Benedetti et al. 2022; Ricotta et al. 2023), *C. angustifolium* can rapidly colonize soils following vegetation removal (Dunnett and Willis 2000), allowing it to expand at the expense of slower‐growing ericoid shrubs (Damgaard et al. 2024).

Species adapted to moist‐ and nutrient‐poor conditions, on the other hand, might be replaced or outcompeted over time. Both *Erica tetralix* (Bannister 1966) and *Sphagnum* mosses (Käärmelahti et al. 2023) are sensitive to shifts in soil moisture and nutrient status. In our study, they were among the species most negatively correlated to road proximity. Similar findings were made in blanket bogs in Spain, where characteristic moss and ericaceous species declined due to hydrological changes following WPP construction (Fraga et al. 2009). To assess the role of nutrient availability in these shifts, we recommend that future studies include direct measurements of soil nutrient concentrations.

### 4.4 Using CSR as a Disturbance Metric

The CSR framework provides a valuable lens for assessing environmental disturbances. Pierce et al. (2017) inferred CSR scores from specific leaf area, leaf dry matter content, and leaf area, allowing for a globally standardized assessment of plant strategies. CSR has been successfully used to show that urbanization reduced the competitive dominance in woody plant communities (Jian and Yang 2024) and that mining and quarrying activities favored stress‐tolerators (Fazlioglu et al. 2021). In our study, CSR was sensitive enough to show that WPP development increased ruderality near roads in peatlands.

However, the interpretive power of CSR as a disturbance metric has limitations. First, wet and nutrient‐poor environments tend to support physiologically specialized plants with inherently stress‐tolerant strategies, resulting in a limited range of CSR scores in our study. Second, some wetland specialists like *Drosera rotundifolia* have high R scores, despite occurring in stress‐dominated communities. At our sites, *D. rotundifolia* occurred mostly far from roads, indicating that high ruderality does not necessarily reflect a positive response to road disturbance. Further, global CSR scores may not accurately reflect functional behavior under regional conditions. For example, *Vaccinium myrtillus* has an R score of 0.4, suggesting moderate investment in rapid reproduction and disturbance‐driven recruitment. In Norway, however, *V. myrtillus* primarily reproduces clonally rather than by seed, which limits its ability to exploit vegetation gaps in the way ruderal species typically would.

Reducing CSR to a function of specific leaf area, leaf dry matter content, and leaf area might miss other traits that play a role in disturbance response. For example, below‐ground bud bank size is increasingly recognized as a key determinant of post‐disturbance regeneration, as large bud banks enable rapid recovery through clonal resprouting (Ott et al. 2019). Similarly, seed size and shape can play a role in disturbance responses, as large and non‐spherical seeds were linked to less persistent seed banks (Wang et al. 2024). Alternatively, disturbance could be assessed through shifts in species commonness at larger scales. As infrastructure‐related disturbances favor generalist‐ or ruderal species, species composition across plots may converge at the expense of rare or specialized species (Mckinney and Lockwood 1999; Yalçinalp et al. 2023). Monitoring such homogenization would provide an additional tool to evaluate disturbance impacts.

### 4.5 Implications for Future Management

Here we demonstrate that the impacts of road construction for WPP development extend well beyond the road itself. Although roads were usually only 8 m wide, changes in plant community composition extended up to 10.8 m into adjacent vegetation on both sides. This effectively increases the footprint of roads by at least two to three times. To reconcile wind energy development with the need for biodiversity conservation, the impacted area should be minimized. This can be done by avoiding sensitive vegetation, optimizing road alignment to reduce the total road area, reducing the construction area to a minimum, and limiting soil disturbance through the use of specialized vehicles (Gallagher et al. 2026; Seifert et al. 2025). In addition, post‐operational site restoration might be required to support vegetation recovery and mitigate long‐term effects of roadbuilding.

## 5 Conclusion

In this study, we investigated how WPP development impacts plant community composition and function at three sites in Norway. We found that plants with ruderal strategies were favored along roads at the expense of stress‐tolerators. This led to a shift in plant community composition even decades after WPP construction. While this shift was confined to road edges, it increased the amount of vegetation that was either lost or significantly altered due to roads at least two‐fold. Based on the findings we recommend that mitigation measures aim at reducing road impacts by planning them carefully and minimizing construction areas. Overall, this could help to protect sensitive plant communities and ensure that wind energy development does not only serve the need for renewable energy production but also aligns with biodiversity conservation goals.

## Author Contributions

**Lukas Seifert:** conceptualization (equal), data curation (equal), formal analysis (equal), investigation (equal), methodology (equal), project administration (lead), supervision (supporting), visualization (equal), writing – original draft (lead), writing – review and editing (equal). **Katrine Sivertsen:** conceptualization (equal), data curation (equal), formal analysis (equal), investigation (equal), methodology (equal), visualization (equal), writing – original draft (supporting), writing – review and editing (equal). **Audun Rugstad:** data curation (supporting), formal analysis (equal), methodology (supporting), visualization (equal), writing – original draft (supporting), writing – review and editing (equal). **Bente J. Graae:** conceptualization (equal), funding acquisition (equal), methodology (equal), supervision (lead), writing – review and editing (equal). **Roel May:** conceptualization (equal), funding acquisition (equal), methodology (equal), supervision (supporting), writing – review and editing (equal). **Dagmar Hagen:** conceptualization (equal), methodology (equal), supervision (supporting), writing – review and editing (equal).

## Funding

This work was supported by Norges Forskningsråd, 321954.

## Conflicts of Interest

The authors declare no conflicts of interest.

## Supporting information


**Figure A1.** Relationship between community‐weighted mean (CWM) ruderality and community‐weighted mean Ellenberg indicator values (EIVs) for moisture (F), light (L), nutrients (N), and reactivity (R). Each point represents a vegetation plot (*n* = 270), with ruderality calculated from vascular plant species only, while EIVs were calculated using all recorded species. Solid lines show linear regression fits with 95% confidence intervals.
**Figure A2**. Species‐specific coefficients for the effect of distance to road by slope within zone 1 and 2 compared to zone 3.
**Figure A3**. Species‐specific coefficients for the effect of the percentage of substrate cover. Slopes for zone 1 and 2 represent the sum of the “base” slope of the effect of distance to road and its interaction coefficient with zones 1 and 2.
**Figure A4**. Species‐specific coefficients for the effect of site identity comparing Frøya (F4) and Ytre Vikna (Y12) wind power plant to Smøla (S19) wind power plant.
**Table A1**. Species that occurred exclusively in zone 1 and number of plots in which they occurred.

## Data Availability

Data available from the Zenodo repository: https://doi.org/10.5281/zenodo.18001566 (Rugstad 2025).
