# Supplementary material for: Onshore Wind Energy Development Causes Localized but Lasting Shifts in Plant Community Composition and Function
Source: Ecol Evol. 2026 Jun 29;16(7):e73916. doi: 10.1002/ece3.73916 (PMC13314548; doi:10.1002/ece3.73916)
Supplement: Supplementary file 1 — Figure A1. Relationship between community‐weighted mean (CWM) ruderality and community‐weighted mean Ellenberg indicator values (EIVs) for moisture (F), light (L), nutrients (N), and reactivity (R). Each point represents a vegetation plot (n = 270), with ruderality calculated from vascular plant species only, while EIVs were calculated using all recorded species. Solid lines show linear regression fits with 95% confidence intervals. Figure A2. Species‐specific coefficients for the effect of distance to road by slope within zone 1 and 2 compared to zone 3. Figure A3. Species‐specific coefficients for the effect of the percentage of substrate cover. Slopes for zone 1 and 2 represent the sum of the “base” slope of the effect of distance to road and its interaction coefficient with zones 1 and 2. Figure A4. Species‐specific coefficients for the effect of site identity comparing Frøya (F4) and Ytre Vikna (Y12) wind power plant to Smøla (S19) wind power plant. Table A1. Species that occurred exclusively in zone 1 and number of plots in which they occurred. [file ECE3-16-e73916-s001.docx]

**APPENDIX**


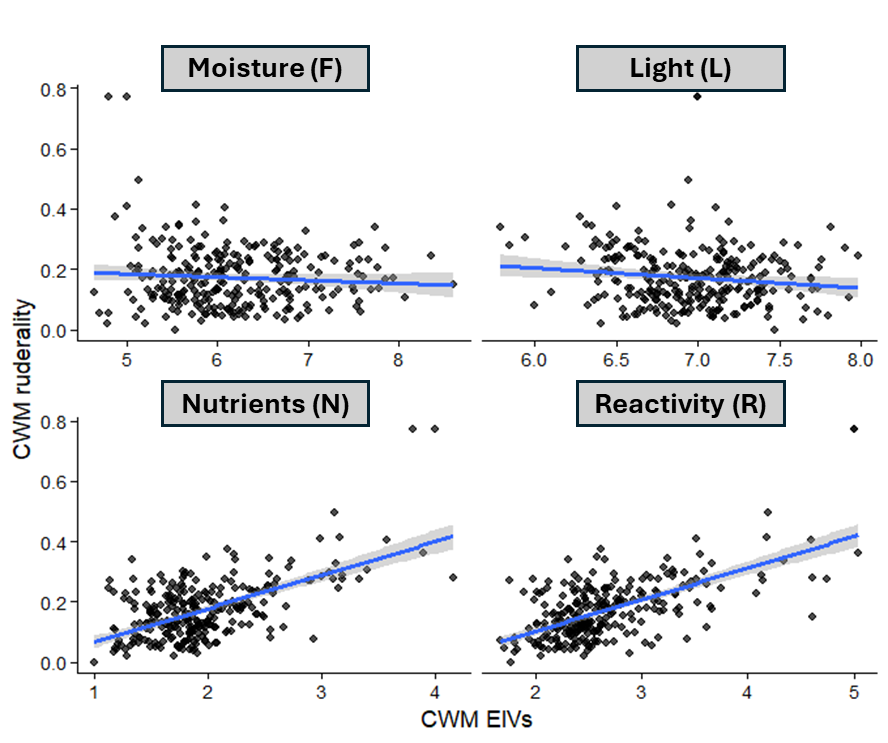


**Figure A1.** Relationship between community-weighted mean (CWM) ruderality and community-weighted mean Ellenberg indicator values (EIVs) for moisture (F), light (L), nutrients (N), and reactivity (R). Each point represents a vegetation plot (n = 270), with ruderality calculated from vascular plant species only, while EIVs were calculated using all recorded species. Solid lines show linear regression fits with 95 % confidence intervals.


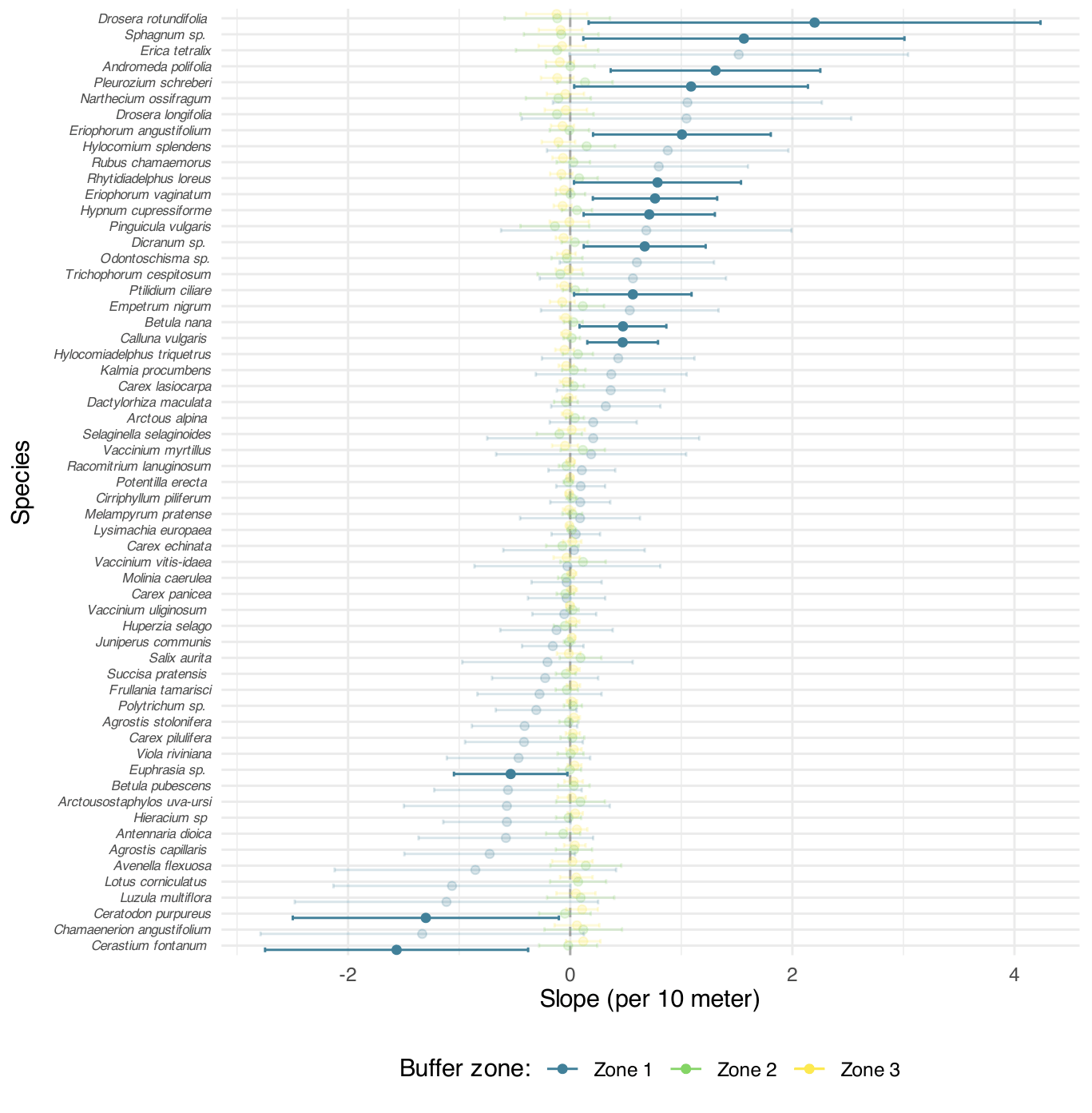


**Figure A2.** Species-specific coefficients for the effect of distance to road by slope within zone 1 and 2 compared to zone 3.


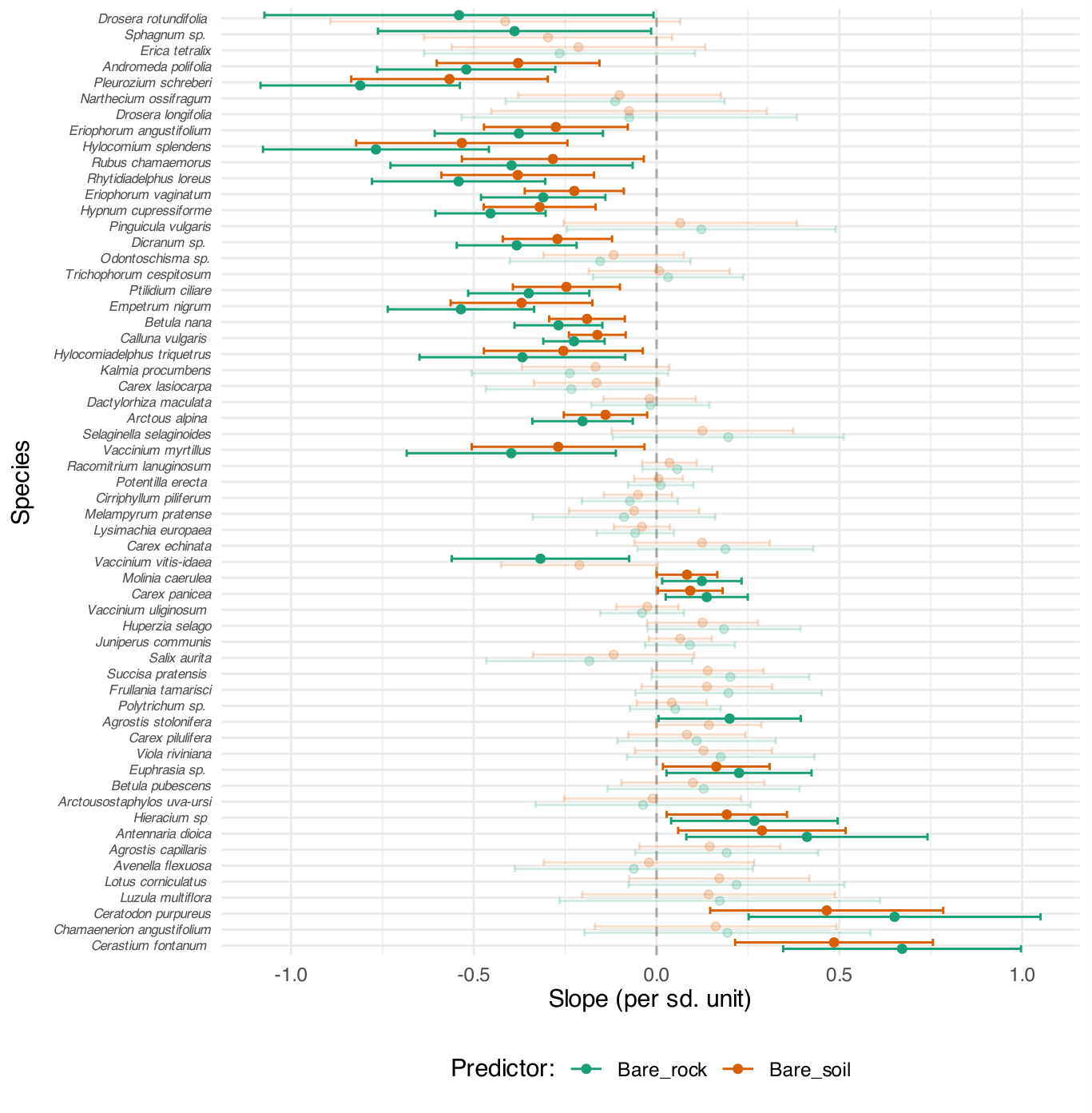


**Figure A3.** Species-specific coefficients for the effect of the percentage of substrate cover. Slopes for zone 1 and 2 represent the sum of the “base” slope of the effect of distance to road and its interaction coefficient with zones 1 and 2.


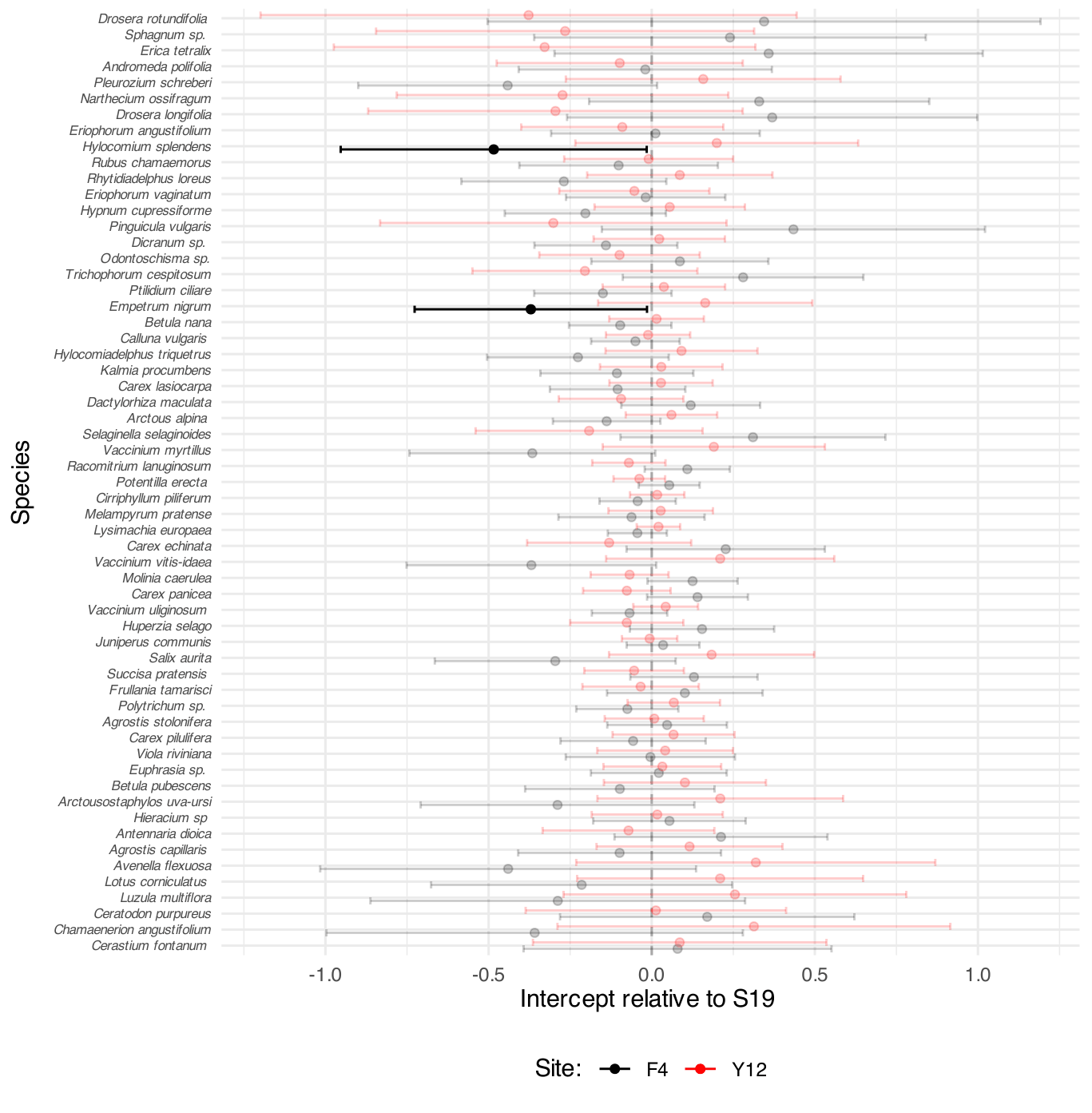


**Figure A4.** Species-specific coefficients for the effect of site identity comparing Frøya (F4) and Ytre Vikna (Y12) wind power plant to Smøla (S19) wind power plant.

**Table A1.** Species that occurred exclusively in zone 1 and number of plots in which they occurred.

| **Species name** | **Occurrence** |
| --- | --- |
| *Athyrium filix‑femina* (L.) Roth | 3 |
| *Aulacomnium palustre* (Hedw.) Schwägr. | 1 |
| *Barbilophozia barbata* (Schmidel ex Schreb.) Loeske | 1 |
| *Calamagrostis epigejos* (L.) Roth | 1 |
| *Carex flava* L. | 3 |
| *Chamaenerion angustifolium* L. Scop. | 20 |
| *Equisetum arvense* L. | 2 |
| *Equisetum pratense* Ehrh. | 1 |
| *Equisetum sylvaticum* L. | 2 |
| *Festuca ovina* L. | 4 |
| *Funaria hygrometrica* Hedw. | 2 |
| *Juncus articulatus* L. | 1 |
| *Juncus effusus* L. | 1 |
| *Lophozia ventricosa* (Dicks.) Dumort. | 1 |
| *Luzula pilosa* (L.) Willd. | 4 |
| *Mnium sp.* | 5 |
| *Picea abies* (L.) H. Karst. | 2 |
| *Rumex acetosa* L. | 2 |
| *Sagina procumbens* L. | 1 |
| *Sagina saginoides* (L.) H. Karst. | 1 |
| *Taraxacum sp.* | 3 |
| *Trifolium pratense* L. | 1 |
| *Trifolium repens* L. | 3 |
| *Tussilago farfara* L. | 4 |
| *Veronica officinalis* L. | 2 |
